# Putative cortical dopamine levels affect cortical recruitment during planning^☆^

**DOI:** 10.1016/j.neuropsychologia.2013.07.016

**Published:** 2013-09

**Authors:** S.J. Fallon, A. Hampshire, C.H. Williams-Gray, R.A. Barker, A.M. Owen

**Affiliations:** a Medical Research Council Cognition and Brain Sciences Unit, Cambridge, CB2 7EF, United Kingdom; b Donders Institute for Brain, Cognition and Behaviour, Radboud University Nijmegen, Nijmegen, Netherlands; c The Brain and Mind Institute, University of Western Ontario, ON, Canada; d Centre for Brain Repair, Department of Clinical Neurosciences, University of Cambridge, CB2 1TN, United Kingdom

**Keywords:** Dopamine, Planning, Catechol *O*-methyltransferase (COMT), Inverted-U, fMRI

## Abstract

Planning, the decomposition of an ultimate goal into a number of sub-goals is critically dependent upon fronto-striatal dopamine (DA) levels. Here, we examined the extent to which the val158met polymorphism in the catechol *O*-methyltransferase (COMT) gene, which is thought to primarily alter cortical DA levels, affects performance and fronto-parietal activity during a planning task (Tower of London). COMT genotype was found to modulate activity in the left superior posterior parietal cortex (SPC) during planning, relative to subtracting, trials. Specifically, left SPC blood oxygenation level-dependent (BOLD) response was reduced in groups with putatively low or high cortical DA levels (COMT homozygotes) relative to those with intermediate cortical DA levels (COMT heterozygotes). These set of results are argued to occur either due to differences in neuronal processing in planning (and perhaps subtracting) caused by the COMT genotype and/or the cognitively heterogeneous nature of the TOL, which allows different cognitive strategies to be used whilst producing indistinguishable behavioural performance in healthy adults. The implications of this result for our understanding of COMT′s effect on cognition in health and disease are discussed.

## Highlights

• Planning performance is influenced by cortical dopamine levels.
• Genetic polymorphisms in the COMT gene affect cortical dopamine levels.
• We examined the effect of COMT genotype of the neural correlates of planning.
• Putatively high and low dopamine led to reduced parietal activity during planning.

## 1 Introduction

Many of the most sought after goals in the world are not immediately attainable. Accordingly, we have had to evolve and develop a capacity to formulate and execute a series of sub-goals whose completion will allow us to achieve our ultimate goal. These behavioural requirements are neatly captured within the Tower of London (TOL) planning task and its cognates (Shallice, 1982). This task has been used extensively to examine the necessary neuroanatomical and neurochemical substrates of planning. Efficient performance on this task has been found to depend upon the integrity of a fronto-striato-parietal network (Carlin et al., 2000; Cazalis et al., 2003; Dockery, Hueckel-Weng, Birbaumer, & Plewnia, 2009; Kaller, Rahm, Spreer, Weiller, & Unterrainer, 2011; Owen, Downes, Sahakian, Polkey, & Robbins, 1990; Shallice, 1982; van den Heuvel et al., 2003). Furthermore, adequate dopaminerergic tone in these areas is also necessary for efficient planning performance. Specifically, pharmacological manipulations and disease states thought to reduce fronto-striatal dopamine (DA) levels have been associated with reduced planning performance (Cools, Stefanova, Barker, Robbins, & Owen, 2002; Lange et al., 1992; Owen et al., 1992; Reeves et al., 2005), whereas increasing DA (and other catecholamines) has been found to improve planning performance (Elliott et al., 1997).

A well-known phenomenon in psychopharmacology is the existence of an inverted-U shape function between DA levels and performance. This phenomenon has been well demonstrated in the case of working memory, where both excess and deficient DAergic stimulation can impair performance (Vijayraghavan, Wang, Birnbaum, Williams, & Arnsten, 2007). Therefore, to the extent that planning depends on cortical functioning, excess cortical DA levels should also impair planning performance. However, there is a dearth of pharmacological substances that can selectively, and safely, modulate cortical DA levels without also affecting striatal DA levels. This makes it difficult to parse out the effects of striatal and cortical DA levels in influencing planning performance. One approach to addressing this problem is to study the effects of genetic polymorphisms that putatively alter cortical DA levels.

The catechol *O*-methyltransferase (COMT) enzyme is thought to have a relatively selective role in regulating cortical DA levels, given that pharmacological or genetic inhibition of this enzyme has been found to have little effect on striatal DA or cortical noradrenaline levels (Tunbridge, Burnet, Sodhi, & Harrison, 2004; Yavich, Forsberg, Karayiorgou, Gogos, & Mannisto, 2007). A single nucleotide polymorphism (SNP), which involves the substitution of valine for methionine at amino acid 158 in membrane bound-COMT (val158met), the dominant form of the enzyme expressed in the brain, plays a key role in determining the enzyme′s activity. Val homozygotes show 40% higher COMT activity compared to Met homozygotes, and thus putatively have lower cortical (PFC) DA levels, whereas heterozygotes are thought to have intermediate levels (Chen et al., 2004). In addition, COMT genotype has also been found to modulate the level of D_1_ receptors throughout the cortex (Slifstein et al., 2008). Thus, the val158met COMT polymorphism provides us with an experimental window into the effects that putative differences in cortical, or at least non-striatal, DA levels, within their normal physiological range, have on cognition in-vivo. In line with this, several studies have reported that Met homozygotes tend to outperform Val homozygotes on tests of working memory and attention, a difference thought to relate to their putatively higher levels of cortical DA (Bertolino et al., 2006; Egan et al., 2001; Meyer-Lindenberg et al., 2006). Moreover, COMT genotype has been found to be an important predictor of planning performance and its concomitant BOLD signal in fronto-parietal areas in patients with Parkinson′s disease (PD: (Foltynie et al., 2004; Hoogland et al., 2010; Williams-Gray et al., 2009; Williams-Gray, Hampshire, Barker, & Owen, 2008; Williams-Gray, Hampshire, Robbins, Owen, & Barker, 2007). More specifically, PD Val homozygotes have been found to outperform PD Met homozygotes. The directionality of this result was explained in terms of the hypothetical DAergic overdosing that is thought to occur in the frontal cortex in early PD in compensation for striatal DA depletion (Bruck, Aalto, Nurmi, Bergman, & Rinne, 2005; Kaasinen et al., 2001; Rakshi et al., 1999), and is supported by the recent finding of increased presynaptic DA in frontal regions in PD Met homozygotes relative to PD Val homozygotes (Wu et al., 2012). However, at present, it is unclear whether the effect of the val158met polymorphism on planning performance is unique to PD patients or is also present within healthy older adults free of parkinsonism. Recently, we reported that the COMT val158met polymorphism modulated attentional performance in healthy older adults in the opposite direction to that observed in PD patients (Fallon, Williams-Gray, Barker, Owen, & Hampshire, 2012). Thus, the COMT val158met polymorphism has differential effects on cognition according to disease status. However, whether the COMT val158met polymorphism modulates planning performance and its neural correlates in a healthly age-matched control group (compared to the PD patients) has yet to be investigated. A recent study in younger adults (mean age=43) found no evidence that COMT genotype modulates behavioural performance or the BOLD response during planning (Stokes, Rhodes, Grasby, & Mehta, 2011). However, DA levels are not static across the lifespan (Backman, Nyberg, Lindenberger, Li, & Farde, 2006; Diamond, 2007; Kaasinen & Rinne, 2002), an effect that is paralleled by the non-linear manner in which COMT modulates cognitive function in different age groups, with the relative performance of each COMT val158met genotype group changing with increasing age (Dumontheil et al., 2011; Harris et al., 2005; Smith & Boettiger, 2012). Thus, it is possible that effects may be present within older adults that are not present in younger adults. Furthermore, consistent with the idea that there is a non-linear relationship between DA levels and behaviour, there may also be a non-linear relationship between COMT activity, as determined by the val158met polymorphism, and behaviour. Indeed, such a relationship has been reported in the case of verbal IQ (Harris et al., 2005). Therefore, this study sought to evaluate whether there is a linear or non-linear relationship between putative DA levels (COMT genotype) and planning performance, and its neural correlates as measured by functional magnetic resonance imaging, in a group of healthy older adults.

## 2 Method

### 2.1 Participants

This study conformed to the Helsinki declaration of 1975 and was approved by the local ethics committee (Suffolk Local Research Ethics Committee number 05/Q102/169). All participants gave written informed consent prior to taking part in this study. An initial sample of 80 participants was recruited to take part in this study from a panel of genotyped older adults. The genotype frequencies and demographics of these participants are presented in Supplementary Fig. 1. These participants completed a variety of neuropsychological tests (see below). From this initial sample, 52 participants agreed to take part in the fMRI study. Results from these 52 participants have previously been reported in the case of attentional control (Fallon et al., 2012) and a voxel based morphometry (VBM) study (Rowe et al., 2010). All participants were evaluated with the Mini Mental State Examination (MMSE; (Folstein, Folstein, & McHugh, 1975), Beck Depression Inventory (BDI; Beck, Epstein, Brown, & Steer, 1988) and National Adult Reading Test (NART; Nelson, 1982). Participants were healthy older adults and were selected to be of comparable age (50<80) to the PD patients tested by Williams-Gray et al. (2007), displaying no evidence of neurological disease on evaluation by a neurologist (CWG), no overt depression (BDI score<15) and no overt signs of dementia (MMSE>25). The demographic data for the subjects in the fMRI study are presented in Table 1. DNA was extracted from peripheral blood samples and genotyping for the COMT val158met polymorphism (SNP rs4680) was performed using a Taqman allelic discrimination assay on a 7900HT Sequence Detection System (Applied Biosystems) using standard protocols (see Williams-Gray et al., 2007).

One-way ANOVAs revealed no significant differences between the different COMT genotype groups (in the fMRI study) in terms of age, *F*<1, *p*>0.05, BDI score, *F*<1, *p*>0.05, MMSE score, *F*(2,51)=1.4, *p*>0.05 or NART IQ score, *F*(2,51)=1.8, *p*>0.05. A chi-squared test between gender and genotype found no differences in the gender ratios between the genotype groups, *χ*^2^=1.3, *p*>0.05.

#### 2.1.1 Neuropsychological tests

To provide a broader perspective on the effect COMT genotype has on an individual's cognitive phenotype, a larger sample of healthy volunteers performed several tests from the Cambridge Neuropsychological Test Automated Battery (CANTAB). These tests included Pattern Recognition Memory (PRM), Spatial Recognition Memory and Paired Associates Learning (PAL; all tests described in (Sahakian et al., 1988). Finally, a behavioural version of ‘one-touch’ version of Tower of London (TOL) was also measured (Owen et al., 1995). These tasks have been described extensively elsewhere, therefore only brief descriptions will be given here. In the PRM task, participants viewed series of 12 patterns and had to correctly identify these patterns in a subsequent memory test. Similarly, for the SRM, participants viewed a series of spatial locations that had to be subsequently identified. The main behavioural metric in both tasks was accuracy (% correct). In the PAL, participants were presented with a an array of 6 boxes. Over successive trials, of varying difficulty, participants were shown the ‘contents’ of each box. Participants had to remember which pattern appeared in which box. The main behavioural measure assessed was the number of participants successively completing the six-shape level (where 6 images had to be paired with 6 locations). The ‘one touch’ TOL task is identical to the planning task used in the fMRI study (see below for more details) except that, in the behavioural version, there were harder problems (5 move problems). There were 14 experimental trials. Accuracy (% correct) and planning latency served as behavioural output measures.

### 2.2 fMRI experimental design and procedure

**Fig. 1:**
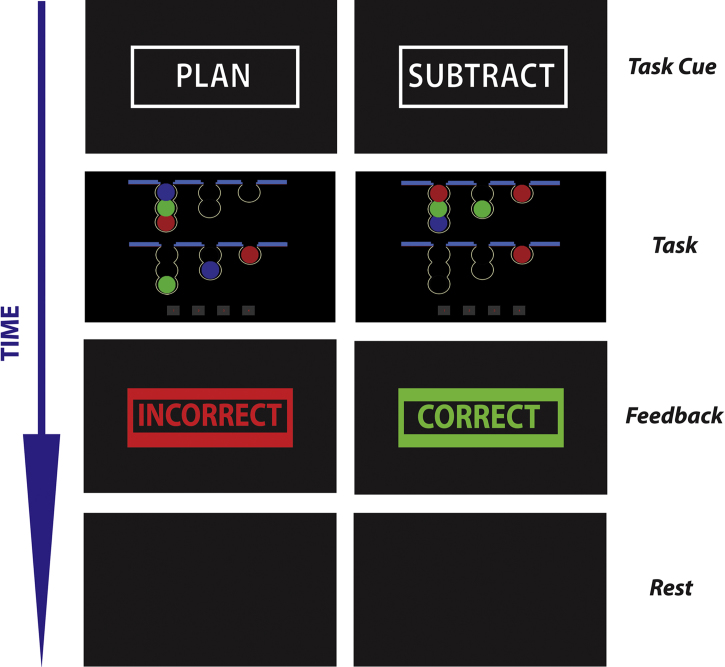
An illustration of a typical series of trials. Plan (left column) and Subtract (right column) trials alternated between each other. Plan and subtract trials were separated by a ‘rest’ period (jittered to last between 5 and 15 s). On each trial participants were cued to either plan or subtract. After making a response, they then received feedback.

The one-touch Tower of London (TOL) task has been extensively described elsewhere (Owen et al., 1995; Williams-Gray et al., 2007). In this version of the task, there were two alternating conditions: planning and subtracting. In both conditions participants were presented with two arrays of balls at the top and bottom of the screen (see Fig. 1). In the planning condition participants had to mentally rearrange the balls on the bottom half of the display (Start state) so as to match the top half of the display (Goal state). They then indicated the minimum number of “moves” that would be required to go from the start state to the goal state using a custom built button box placed under the right hand. The mapping of buttons to numbers was displayed throughout the trial at the bottom of the screen. Participants were informed that each pocket could only “hold” a certain number of balls. For example, the left pocket could only “hold” a maximum of 3 balls, the middle pocket 2 balls and the right pocket 1 ball. Furthermore, participants were told that they could not move a ball that was currently sitting beneath another ball. In this condition, difficulty was manipulated by varying the number of (imagined) moves that had to be made to go from start state to the goal state. There were three levels of difficulty (problems that required 2–4 moves). In the subtracting control condition, participants saw a similar visual display to the planning condition. However, instead of having to work out how to match the two configurations of balls, participants had to count the number of balls in the top half of the screen and subtract that number from the total number of balls in the bottom half of the screen and indicate the result using the button box. In this condition, difficulty was manipulated by varying the difference in the number of balls between the top and bottom half of the display (2–4). In both conditions participants received feedback about the correctness of their response via presentation of the word ‘Correct’ in the centre of the screen in green bold font or the word “Incorrect” in red at the centre of the screen. Thus, the two conditions were matched for visual, motoric and motivational requirements. This allowed us to compare the effect that planning had on the BOLD response over and above other non-specific effects. Given that the duration of the planning and subtracting events were response driven, the number of problems completed varied.

Participants gave informed consent prior to taking part in the study and were familiarized with the task. They were trained for 5 min on the task prior to entering the scanner. They were instructed to respond as quickly and accurately as possible. In the scanner, participants were informed about the mappings between the button box and viewed the experiment on a monitor with a 1280×1024 resolution which they saw via a mirror. The task ran for one 10 min session. The viewing distance from the monitor was 90 mm with 37 pixels subtending a visual angle of 1°. After performing this task, participants completed another cognitive task, the results of which have been reported elsewhere (Fallon et al., 2012).

The main behavioural measures on this task were accuracy and response latency. Accuracy and response latency were broken down according to problem difficulty, i.e., how many moves were required in order to solve the problem. Accuracy data were quantified in terms of the proportion of problems that were solved correctly and were arcsine transformed to meet parametric test assumptions. Reaction time data were log transformed. Behavioural data were analysed in SPSS 16.0 (SPSS, Chicago, IL).

### 2.3 fMRI analysis

#### 2.3.1 fMRI scanning protocol

Data were acquired on a 3T Siemans Tim Trio MRI scanner. Imaging parameters were as follows: TR=2 s, TE=30 ms, FOV=192. 32 Slices (3 mm thick with a 1 mm interslice gap) were acquired in a descending order with slices angled down away from the orbits. Three hundred and thirty images, with a within plane resolution of 3.0×3.0 mm, were acquired. The first 10 volumes (20 s) were discarded to allow for equilibrium of the signal.

#### 2.3.2 Pre-processing

fMRI data were analyzed using SPM5 (Wellcome Department of Imaging Neuroscience, UCL, UK). Preprocessing was implemented using an automated analysis script, aa version 1 (cusacklab.org). The individual preprocessing steps were performed in the following order. First, functional images were realigned to the first image acquired according to a 6 parameter rigid body transformation. Slice Timing correction was then applied to the data (“shifting” the timecourse of activation to the first slice in an image). The structural image was then co-registered with the mean functional image. The SPM5 combined segmentation/normalization procedure (Ashburner & Friston, 2005) was then performed, normalizing the structural image to the standard imaging template (MNI), and the resultant transform was applied to the functional images. The grey matter mask generated from this analysis was used to calculate total grey matter volume to use as a covariate in subsequent analyses. Finally, the data was smoothed using an 8 mm full width at half maximum Gaussian kernel.

#### 2.3.3 First level modeling

Data were analyzed according to the “summary statistics approach” (Holmes & Friston, 1998) in SPM5 in which a summary statistic (beta weight) generated from a separate general linear model (GLM) for each participant is passed to a second-level GLM, in order to facilitate group inferences. The first-level design matrix comprised six task-related regressors, each of which was generated by convolving the onsets and durations for events of interest with a canonical haemodynamic response function. The task regressors comprised of 3 planning events and 3 subtracting events (one for each level of difficulty: 2 move problems, 3 move problems and 4 move problems). Onsets were determined according to the time that the planning or subtracting problems were presented to the participant, whereas durations were determined by the latency of participants′ responses. Following (Lund, Norgaard, Rostrup, Rowe, & Paulson, 2005), we included 24 movement parameters in our design matrix as regressors of no interest. These include the six parameters used to realign the functional images to each other (corresponding to rotations and translations within the *x*, *y*, and *z* planes), as well as the first derivatives of these terms, the quadratic function of these and, to account for spin history effects (Friston, Williams, Howard, Frackowiak, & Turner, 1996), the 6 transformation parameters used to realign the previously acquired volume. Finally, the data were high pass filtered (128 s) and serial correlations present in fMRI data were adjusted using an AR(1) model.

For each participant, a “Planning minus Subtracting” contrast image was formed by subtracting the beta weights found for the subtracting (control) conditions (2–4 problems) from the beta weights found for the planning conditions (2–4 move problems). This image was passed to the second-level.

#### 2.3.4 Second-level analysis

Linear and non-linear effects of COMT on planning activity (planning minus subtracting contrast) were assessed at the second level using multiple linear regression in SPM5. Two orthogonal covariates were entered into the design matrix. The first accessed whether there was a linear effect of COMT (number of Val alleles), and the second corresponded to their being a non-linear effect, inverted-U shape function, between COMT activity and activation (2 for COMT heterozygotes and -1 for COMT homozygotes). Both covariates were mean centred. Analyzing the data in this fashion allows us to examine linear effect of COMT whilst also accounting for non-linear effects of COMT (and vice-versa).

## 3 Results

### 3.1 Neuropsychological performance

There was no significant effect of COMT genotype on performance for the PRM, SRM, PAL or out-of-scanner TOL performance (*p’*s>0.05; see Supplementary Figs. 1 and 2). Thus there were no gross differences in neuropsychological performance between the COMT genotype groups.

### 3.2 In-scanner behavioural results

One participant was excluded from all behavioural and fMRI analysis due to apparent striatal calcification. Differences in planning performance, both for accuracy and response latency, were assessed using a mixed ANOVA with repeated measures on task type (planning versus control condition) and problem difficulty (2–4 move problems) with COMT genotype as between-subject variable. For planning accuracy (see Fig. 3), there was a significant main effect of condition, *F*(1,48)=55.39, *p*<0.05, in the direction of participants being less accurate on planning trials. There was a slight trend towards a main effect of difficulty, *F*(2,96)=2.56, *p*=0.08, but no significant interaction between condition and difficulty (*F*<1). There was no main effect of genotype, genotype by difficulty interaction or interaction between genotype and condition (*F*s<1). Finally, there was no significant three-way interaction between condition, difficulty and genotype *F*(4,96)=1.88, *p*>0.05. These results show that there were no significant effects of COMT genotype in terms of planning or subtracting accuracy.

Differences in response latency (Fig. 3, top right) during planning trials were examined using the same statistical model. There was a main effect of condition, *F*(1,48)=492.80, *p*<0.05, and difficulty, *F*(2,96)=14.52, *p*<0.05. There was also a significant interaction between condition and difficulty, *F*(2,96)=19.56, *p*<0.05. Simple main effects analysis revealed that whilst response latencies significantly differed for each difficulty level in the planning condition, *F*(2,47)=26.01, *p*<0.05, there was no such difference in the subtracting condition (*F*<1). There was no significant interaction between difficulty and genotype, *F*(4,96)=1.28, *p*>0.05. No other effects were significant (*F*s<1).

### 3.3 Functional neuroimaging results

**Fig. 2:**
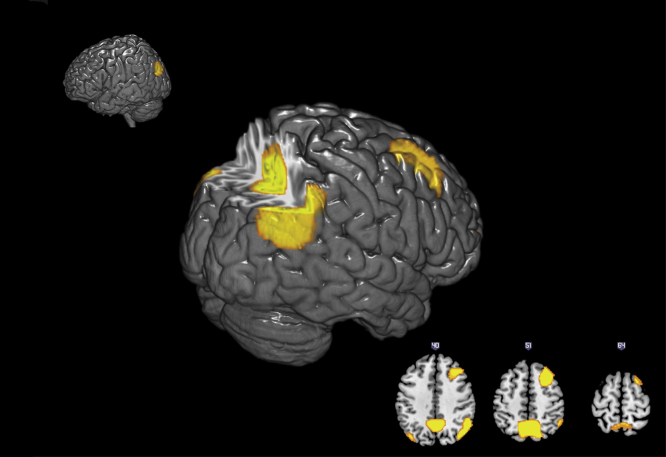
*All figures*: A statistical parametric map (SPM) of areas showing significantly (*p*<0.05 Family wise Error corrected) increased BOLD signal during planning compared to subtracting. *Top left*: left hemisphere response. *Centre figure*: right hemisphere activation with a posterior section removed. *Bottom right*: Three horizontal sections of the same statistical contrast.

**Fig. 3:**
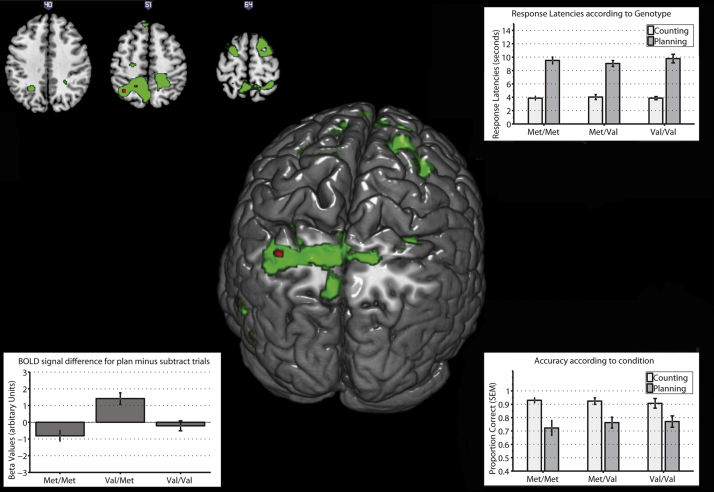
*Centre and top left*: SPMs of significantly increased BOLD signal for COMT heterozygotes compared to COMT homozygotes (quadratic effect of COMT genotype) during planning relative to subtracting. Areas in green represent significantly increased activity at *p*<0.001(uncorrected), whereas areas in red represent whole-brain corrected (FWE<0.05) activity. *Bottom left*: parameter estimates (beta values) for each genotype group in the left superior parietal cortex (SPC; 6 mm sphere around *X*=−36, *Y*=−56, *Z*=52). *Top right*: response latencies for planning and subtracting trials according to COMT genotype groups. *Bottom right*: accuracy for planning and subtracting trials. For all graphs error bars correspond to Standard Error of the Mean (SEM).

Examining the group-level activation for the planning minus subtracting contrast revealed a significant increase in BOLD signal in frontal and parietal areas (see Fig. 2; see Supplementary Fig. 3 for significant regions). No voxels were found to have a significant positive or negative linear relationship to the number of Val alleles at the 0.05 (FWE-corrected) threshold, even at the more liberal 0.001 (uncorrected) threshold. However, voxels in the left superior parietal cortex (SPC; [*x*=−36 *y*=−56 *z*=52]) and left-medial superior parietal cortex (mSPC; [*x*=−20 *y*=−50 *z*=52]) were found to have a significant (0.05, FWE-corrected) non-linear (inverted-U shape) relationship to the number of Val alleles (see Fig. 3; Supplementary Figs. 5 and 6). That is, COMT heterozygotes showed increased BOLD signal in these regions compared to COMT homozygotes. This effect of genotype persisted even when age, gender, total grey matter volume and NART IQ were included as covariates. Supplementary analyses examined whether the effect of COMT varied according to difficulty. There was no significant interaction between COMT and difficulty (Supplementary Fig. 7).

For the inverse contrast (homozygotes minus heterozygotes), there was no significant difference in BOLD signal, even at the liberal threshold of *p*<0.01 (uncorrected).

### 3.4 BOLD response and performance

In order to explore the behavioural implications of activation in the left SPC (*x*=−36 *y*=−56 *z*=52), the level of activation in this region (extracted beta values for 6 mm spherical ROIS) was correlated with planning performance (both in terms of accuracy and reaction time), separately for each COMT genotype group. The left mSPC was excluded from this analysis in order to limit the number of comparisons. As can be seen from (Table 2), Left SPC activity was positively correlated with planning response latency only in Val/Val participants (*rho*=.50, *p*<0.05), i.e., greater left SPC activity was associated with *slower* reaction times in val homozygotes. In contrast, none of the other correlations were significant (see Table 2).

## 4 Discussion

This study has reinforced our knowledge of the neural substrates of planning and has added to our understanding of how putative differences in cortical DA levels modulate neuronal (BOLD) signals in healthy older adults. In line with previous studies, planning was found to engage a broad swath of fronto-parietal areas (Beauchamp, Dagher, Aston, & Doyon, 2003; Newman, Carpenter, Varma, & Just, 2003; Rowe, Owen, Johnsrude, & Passingham, 2001; van den Heuvel et al., 2003; Wagner, Koch, Reichenbach, Sauer, & Schlosser, 2006). Interestingly, despite this wide-scale fronto-parietal activation, only the left SPC was modulated by COMT genotype. More specifically, activity in the left SPC was found to correlate with Val-allele load in a non-linear manner. Given that there is thought to be a linear relationship between Val allele load and DA levels (Chen et al., 2004), this result suggests that having either low or high cortical DA levels leads to diminished recruitment of the left SPC during planning (relative to subtracting).

Although, the val158met polymorphism is thought to predominantly modulate DA levels in the PFC, the parietal difference is consistent with previous findings that this polymorphism modulates D_1_ DA receptor density throughout the cortex (Slifstein et al., 2008) and parietal BOLD signal during cognitive tasks (de Frias et al. 2010; Williams-Gray et al., 2008; Williams-Gray et al., 2007). The present study, however, represent a slight departure from the majority of previously published work on COMT modulation of behavioural and neuronal functioning. These studies have reported that Met homozygotes tend to outperform Val homozygous on working memory tasks and show more efficient neural (BOLD) responses during these tasks (de Frias et al., 2005; Tan, Callicott, & Weinberger, 2007; Winterer et al., 2006, 2006). Furthermore, this pattern of results is reversed by pharmacological manipulations that increase PFC DA levels (Apud et al., 2007; Farrell, Tunbridge, Braeutigam, & Harrison, 2012; Mattay et al., 2003). These findings suggest that Met homozygotes are positioned at the apex of an inverted-U shape function that links DA with both performance and neuronal processing. However, in this study, it would appear that it is COMT heterozygotes who sit at the apex of an inverted-U shaped function. Previous investigations into COMT′s neuronal effects have found that there is a positive relationship between BOLD response and signal-to-noise ratio (SNR; extensively reviewed in Winterer et al., 2006). Therefore, one explanation for the increased BOLD response in COMT heterozygotes during planning may reflect optimal neuronal functioning, i.e., enhanced SNR. However, this study uncovered a complicated, genotype-specific, relationship between BOLD activation and planning performance. That is, increased BOLD activation in the left SPC was associated with slower planning times in val homozygotes, i.e., increased neuronal inefficiency, but no such effect was present in the other COMT genotype groups. Thus, the results suggest that increased activity in the left SPC is malapdative for val homozygotes, but adaptive (or have no relationship) for other COMT genotype groups. Therefore, while this study has demonstrated that there is a non-linear relationship between between putative DA levels and BOLD signal, it can only offer limited support for the idea that there is an inverted-U shape relationship cortical between DA levels and neural activity during planning. Further evidence, perhaps incorporating appropriate pharmacological manipulations and direct measurement of cerebral DA levels using PET, is needed in order to provide firmer evidence for the existence of an inverted-U shape function between DA and neural activation during planning.

Alternatively, but not mutually exclusively, it is also possible that BOLD signal differences (in the absence of any behavioural effects) may result from individual differences in cortical recruitment. That is, while COMT heterozygotes preferentially recruit the left SPC for planning, COMT homozygotes preferentially recruit the left SPC for subtracting. This interpretation is supported by the fact that whereas COMT heterozygotes show increased activity in the left SPC for planning minus subtracting, the reverse is the case for COMT heterozygotes (Fig. S3). Under this framework, Fig. 2 can be seen as representing areas that are commonly recruited to support planning, whereas Fig. 3 can be seen as showing areas where there are individual differences in cortical recruitment for planning relative to subtracting. However, why there would be such a difference in recruitment is unclear. Importantly, given that we found an interaction between COMT genotype and task, it is unlikely that this difference in cortical recruitment results from differences in generic physiological changes, e.g., altered neurovascular coupling. Rather, one functional explanation for this difference is that there is altered connectivity between the frontal regions and the left SPC. Indeed, a recent study found that there were intrinsic differences in connectivity between frontal regions and parietal regions, even when participants are not engaged in a task (Tunbridge, Farrell, Harrison, & Mackay, 2013). Thus, the supposition of task demands on this region may result is very different patterns of activation. However, this does not explain why there were differences in cortical recruitment for the two tasks.

One explanation for this difference is the use of different, but equally effective, strategies to perform both the planning and subtracting tasks. This interpretation is consistent with the differential brain-behaviour relationship according to COMT genotype. The finding of differently signed correlations with performance in different groups has resonance with previous working memory studies in healthy older adults (Grady et al., 1998; Rypma & D′Esposito, 2000). Such effects are usually thought to arise from the use of different strategies to complete a task. Therefore, COMT genotype′s effect on neural activity (BOLD response) may result from qualitatively different ways of performing the TOL, but which lead to the same performance level. In line with this, several studies have highlighted the heterogeneous nature of the planning problems that occur in the TOL, and that different types of planning problems require the use of different strategies and preferentially recruit distinct cortical areas (Kaller et al., 2011; Newman et al., 2003). As such, each behavioural sub-component involved in planning may be differentially sensitive to modulation by COMT genotype. One of the testable hypotheses generated by this explanation is that planning and subtracting are accomplished by qualitatively different networks in different COMT genotype groups (see (Tan et al., 2007) for similar arguments in the case of COMT and working memory). This idea is also consistent with previous studies that have found that dopaminergic manipulations can alter the relationship between certain brain regions and performance during planning (Nagano-Saito, Liu, Doyon, & Dagher, 2009). This hypothesis can most directly be addressed by a transcranial magnetic stimuluation (TMS) study that examines whether the functional consequences of TMS-induced modulations of frontal or parietal regions differ according to COMT genotype. Indeed, a recent study that, prior to an fMRI scan, applied off-line low frequency TMS to the left dorsolateral PFC found an altered BOLD signal in a left superior parietal region in close proximity to the left SPC region found here (van den Heuvel, Van Gorsel, Veltman, & Van Der Werf, 2013). Thus differential consequences of fronto-parietal modulation may ensue in different COMT genotype groups.

In a similar vein, recent computational modeling of DA′s effect on PFC microcircuits has suggested that the val158met polymorphism may influence cognition in an orthogonal manner to that assessed in the TOL. That is, COMT may modulate the balance between cognitive stability and cognitive flexibility (Durstewitz & Seamans, 2008). Given that the TOL is dependent upon both cognitive stability (e.g. maintaining the number of moves made) and cognitive flexibility (e.g. exploring new solutions), any effect COMT genotype has on the balance between cognitive stability/flexibility will not manifest itself on TOL performance. This contention is supported by a large-scale study of cognitive function in healthy older adults that found that planning performance and attentional flexibility load on separate cognitive factors (Robbins et al., 1998). Furthermore, this conclusion goes someway to resolving the discrepancy between our present result and our previous studies. Recently, in the same cohort of healthy older adults tested here, we found that the val158met polymorphism modulates the balance between cognitive stability and flexibility (Fallon et al., 2012). A linear effect of Val allele load was observed, with an increase in Val allele load being associated with enhanced attentional flexibility, but reduced attentional stability. However, in this study the effects of COMT were much more subtle and different in character (non-linear as opposed to linear). Given the preceding discussion, a likely cause for this discrepancy is the failure of the TOL to detect subtle, DA-induced changes in PFC functioning and cognition, i.e., modulating the balance between cognitive stability and cognitive flexibility). This lack of a behavioural effect of COMT genotype on planning performance (seen in both the in-scanner performance and the larger behavioural study) is consistent with the lack of behavioural effects observed in other studies (Stokes et al., 2011). Given that COMT genotype primarily affects cortical DA levels, the lack of a behavioural effect is also consistent with studies that have found that striatal DA levels affect performance on the TOL (Reeves et al., 2007). Thus, cumulatively, the TOL may be ill-suited to examine how COMT genotype affects cognitive phenotype, at least in healthy populations (see below).

The relatively weak effects of COMT genotype on planning performance in healthy older adults stands in stark contrast to the effects of COMT genotype in PD (Williams-Gray et al., 2007, 2009). A formal quantitative comparison between the performance of PD patients and health controls revealed that COMT genotype does affect planning performance to a greater extent in patients than controls (see Supplemental Fig. 8). Thus, COMT genotype affects planning performance in PD patients in ways that are not present in healthy older adults. Previously, COMT genotype was found to predict attentional set-formation, planning efficacy and concomitant BOLD response in fronto-parietal areas in PD patients (Fallon et al., 2012; Williams-Gray et al., 2008, 2007). In both of these tasks, PD Val homozygotes were found to out-perform PD Met homozygotes. The directionality of these results was explained in terms of PD patients being situated on the right-hand limb of an inverted-U shape response function. As mentioned above, COMT modulates attentional flexibility in healthy controls in the opposite manner to that seen in PD patients (Fallon et al., 2012). However, in the case of planning, this study did not find that the val158met polymorphism produced symmetrical effects in PD patients and healthy older adults. Thus, it appears that there is the equivalent of a three-way interaction between disease, genotype and task. It seems likely that unique, disease-specific mechanisms are responsible for generating the discrepancy between the effect COMT has on cognitive function in healthy older adults and PD patients. Amongst the likely list of candidate mechanisms is the presence of numerous co-existing pathologies and PD patient′s DArgic medication. The most well characterized neuropathological marker of PD is degeneration of DArgic midbrain neurons in the substantia nigra (Jellinger, 1987). Interestingly, the val158met polymorphism has also been found to modulate DA levels in the midbrain (Akil et al., 2003; Meyer-Lindenberg et al., 2005). Therefore, this modulation of midbrain DA levels may uniquely affect cognition in PD. Furthermore, val158met genotype has been found to effect PD patient′s response to certain DAergic medications (Corvol et al., 2011). Thus, the Parkinsonian brain may allow COMT genotype to have a greater impact on cognitive function than is the case in healthy older adults. However, it is unclear whether these factors alone can account for this discrepancy between COMT′s effects on planning and attentional control in healthy older adults and COMT′s effects in PD patients. It seems likely that the exact cognitive requirements of the task determine the scope of COMT′s effects. Indeed, PD patients have been found to have specific problems in correctly sequencing the various sub-goals required to solve the planning problems in the TOL (McKinlay et al., 2008) and have been found to show aberrant problem solving strategies on this task (Hodgson, Tiesman, Owen, & Kennard, 2002). Thus, further studies are required in order to elucidate the neurochemical mechanisms involved in COMT′s modulation of planning in PD.

It is the complex and mercurial actions of DA on neuronal networks that give rise to the non-linear relationship between DA levels and cognitive function. This study has found evidence for a non-linear effect of PFC DA levels on neural activation during planning. This difference is likely to occur as a result of each group using different cognitive (and cortical recruitment) strategies to perform the task. As such, this study has added to our knowledge of how COMT (DA) may lead to the formation of different cognitive phenotypes with different strengths and weaknesses (Stein, Newman, Savitz, & Ramesar, 2006). Finally, this study has also revealed that COMT genotype can have effects in patient groups (PD) that are not present (or at least to the same extent) in the healthy population.

## Figures and Tables

**Table 1:** Participants demographics according to COMT genotype.

	Met/Met	Val/Met	Val/Val
Age	63.6 (6.6)	66.2 (7.4)	63.5 (6.9)
MMSE	29.5 (.62)	29.2 (.77)	29.5 (.60)
NART IQ	120.7 (5.7)	123.7 (2.5)	120.8 (6.0)
BDI	4.4 (3.4)	3.2 (3.0)	3.9 (2.7)
Gender(M/F)	7:10	8:7	8:12
*N*	17	15	20

**Table 2:** Pearson (rho) correlations between BOLD signal in the left superior parietal cortex and behavioural performance indices.

Behavioural measure	COMT genotype group	*N*	PA	PRT	Left SPC activity
Planning accuracy (PA: % correct)	Met/Met	17		.36	.01
	Val/Met	14		−.54^⁎^	−.13
	Val/Val	20		−.05	.02

Planning reaction time (PRT)	Met/Met	17			.17
	Val/Met	14			.−34
	Val/Val	20			.50^⁎^

* indicates p < 0.05

## Footnotes

Appendix A Supplementary data associated with this article can be found in the online version at doi:10.1016/j.neuropsychologia.2013.07.016.
