# Supplementary material for: Putative cortical dopamine levels affect cortical recruitment during planning
Source: Neuropsychologia. 2013 Sep;51(11):2194–201. doi: 10.1016/j.neuropsychologia.2013.07.016 (PMC3808120; doi:10.1016/j.neuropsychologia.2013.07.016)
Supplement: Supplementary file 1 — Supplementary material [file mmc1.docx]

**Supplemental figures**

|  | Met/Met | Val/Met | Val/Val |
| --- | --- | --- | --- |
|  |  |  |  |
| Age | 63.6 (6.6) | 66.2 (7.4) | 63.5 (6.9) |
| MMSE | 29.5 (.62) | 29.2 (.77) | 29.5 (.60) |
| NART IQ | 120.7 (5.7) | 123.7 (2.5) | 120.8 (6.0) |
| BDI | 4.4 (3.4) | 3.2 (3.0) | 3.9 (2.7) |
| Gender(M/F) | 7:10 | 8:7 | 8:12 |
| PRM Accuracy (%)  SRM Accuracy (%) | 92.0 (7.1)  80 (6.7) | 92.0 (8.4)  83 (9.5) | 90 (7.4)  80 (8.6) |
| PAL (% pass stage 6) | 86.4 | 70.4 | 77.4 |
| *N* | 22 | 31 | 27 |

Supplemental Figure 1: The demographics and neuropsychological performance for the larger sample of healthy older adults. There was no significant difference between the genotype groups on these measures; all *p*’s > 0.05. MMSE = Mini Mental State Examination; NART = National Adult Reading Test; BDI = Beck Depression Inventory; PRM = Pattern Recognition Memory; SRM = Spatial recognition Memory; PAL = Paired associates learning.


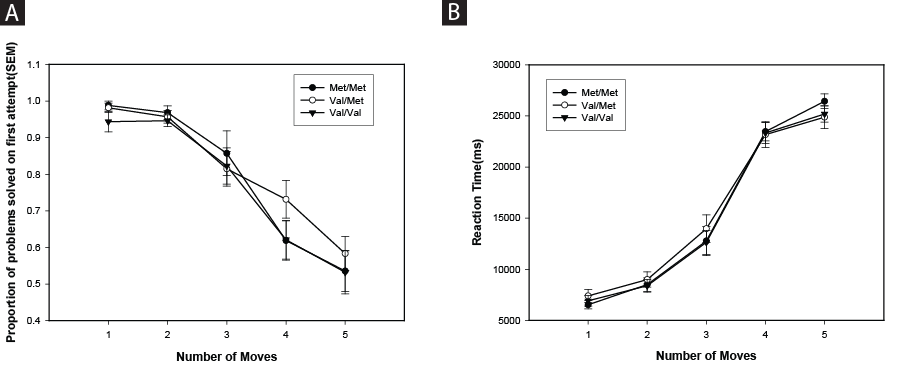


Supplemental Figure 2: Neuropsychological performance as a function of COMT genotype in the larger group. Figures 2A shows accuracy on the out of scanner one touch TOL task as a function of difficulty. Figure 2B shows reaction time for successfully completed TOL problems. There was no main effect of COMT genotype or interaction between COMT and problem difficulty (*F*s<1).

| Region | Cluster Extent (k) | T Score | Z Score | X | Y | Z |
| --- | --- | --- | --- | --- | --- | --- |
| Precuneus | 2321 | 11.01 | 7.73 | 4 | -58 | 48 |
| Right Parietal | 1322 | 10.87 | 7.68 | 46 | -72 | 30 |
| Dorsolateral prefrontal cortex | 1099 | 8.56 | 6.64 | 24 | 18 | 48 |
| Left Parietal | 383 | 8.38 | 6.55 | -42 | -80 | 28 |
| Left Inferior Precuneus | 95 | 6.56 | 5.52 | -16 | -58 | 22 |
| Left Inferior Parietal | 14 | 5.16 | 4.58 | -40 | -54 | 26 |
| Anterior Frontal lobe | 4 | 5.08 | 4.52 | 28 | 60 | 2 |

Supplemental Figure 4: Table of significant activity (P < 0.05 Family Wise Error Corrected) for planning minus Counting Contrast. Only the peak voxels in each cluster are reported.

| Region | Cluster Extent (k) | T Score | Z Score | X | Y | Z |
| --- | --- | --- | --- | --- | --- | --- |
| Left superior parietal lobule | 2768 | 5.07 | 4.52 | -36 | -56 | 52 |
|  |  |  |  |  |  |  |
|  |  | 5.03 | 4.49 | -20 | -50 | 52 |
|  |  |  |  |  |  |  |
|  |  | 4.46 | 4.06 | 20 | -44 | 48 |
|  |  |  |  |  |  |  |
| Right superior frontal gyrus | 452 | 4.08 | 3.76 | 22 | 16 | 62 |
|  |  |  |  |  |  |  |
|  |  | 4.00 | 3.69 | 34 | 4 | 60 |
|  |  |  |  |  |  |  |
|  |  | 3.79 | 3.53 | 20 | -2 | 60 |
|  |  |  |  |  |  |  |
| Right superior orbital gyrus | 97 | 4.05 | 3.74 | 18 | 20 | -10 |
| Left middle frontal gyrus | 265 | 3.97 | 3.68 | -22 | 4 | 60 |
|  |  | 3.76 | 3.51 | -10 | 6 | 70 |
|  |  |  |  |  |  |  |
|  |  | 3.56 | 3.34 | -18 | -4 | 72 |
|  |  |  |  |  |  |  |
| Left middle temporal gyrus | 61 | 3.86 | 3.58 | -56 | -66 | 12 |
|  |  |  |  |  |  |  |
|  |  | 3.34 | 3.15 | -52 | -72 | 6 |
|  |  |  |  |  |  |  |
| Right middle orbital gyrus | 56 | 3.79 | 3.52 | 14 | 36 | 2 |
|  |  |  |  |  |  |  |
|  |  | 3.32 | 3.14 | 12 | 30 | -6 |
|  |  |  |  |  |  |  |
| Left middle frontal gyrus | 43 | 3.67 | 3.43 | -32 | 28 | 30 |

Supplemental Figure 5: Table of significant activity (thresholded at p < 0.001, uncorrected, k > 40) for quadratic effect of COMT genotype (heterzyogotes > homozygotes).

Supplemental Figure 6: Extracted beta values for the left superior parietal cortex (SPC; 6mm sphere around X = -36, Y = -56, Z =52) for planning and counting trials according to COMT genotype. Error bars correspond to Standard Error of the Mea (SEM)

Supplemental Figure 7: Extracted beta values for the left superior parietal cortex (SPC; 6mm sphere around X = -36, Y = -56, Z =52) for planning trials split according to difficulty and COMT genotype. Error bars correspond to Standard Error of the Mea (SEM). There was no main effect of difficulty or interaction between difficulty and genotype (*F*s<1)

Supplemental Figure 8: Participants performance (response latency) for each level of difficulty according to each disease and genotype. Data from Parkinson’s disease patients comes from Williams-Gray et al (2007) and readers are instructed to consult this paper for patient demographics. It should be noted that a factorial 2 (healthy, disease) x 2 (val homozyotes, met homozygotes) ANOVA found that there was a significant interaction between disease and genotype was also a significant interaction between disease and genotype, *F*(1, 64) = 4.38, *p* < 0.05. Simple main effect analysis found that there was a significant effect of genotype in PD patients, *F*(1, 64) = 6.75, *p* < 0.05(as was reported in Williams-Gray et al, 2007), but not in healthy older adults (*F*<1). The absence of a PD COMT heterozygote group prevented us from including COMT heterozygotes in the analysis.
